# Protection against discrimination in national dementia guideline recommendations: A systematic review

**DOI:** 10.1371/journal.pmed.1003860

**Published:** 2022-01-11

**Authors:** Tiffeny James, Naaheed Mukadam, Andrew Sommerlad, Hossein Rostami Pour, Melanie Knowles, Ignacia Azocar, Gill Livingston

**Affiliations:** 1 Division of Psychiatry, University College London, London, United Kingdom; 2 South London and Maudsley NHS Foundation Trust, London, United Kingdom; 3 Camden and Islington NHS Foundation Trust, London, United Kingdom; University of New South Wales, AUSTRALIA

## Abstract

**Background:**

National dementia guidelines provide recommendations about the most effective approaches to diagnosis and interventions. Guidelines can improve care, but some groups such as people with minority characteristics may be disadvantaged if recommended approaches are the same for everyone. It is not known if dementia guidelines address specific needs related to patient characteristics. The objectives of this review are to identify which countries have national guidelines for dementia and synthesise recommendations relating to protected characteristics, as defined in the UK Equality Act 2010: age, disability, gender identity, marriage and civil partnership, pregnancy and maternity, race, religion or belief, sex, and sexual orientation.

**Methods and findings:**

We searched CINAHL, PsycINFO, and Medline databases and the Guideline International Network library from inception to March 4, 2020, for dementia guidelines in any language. We also searched, between April and September 2020, Google and the national health websites of all 196 countries in English and in each country’s official languages. To be included, guidelines had to provide recommendations about dementia, which were expected to be followed by healthcare workers and be approved at a national policy level. We rated quality according to the iCAHE guideline quality checklist. We provide a narrative synthesis of recommendations identified for each protected characteristic, prioritising those from higher-quality guidelines. Forty-six guidelines from 44 countries met our criteria, of which 18 were rated as higher quality. Most guidelines (39/46; 85%) made at least one reference to protected characteristics, and we identified recommendations relating to age, disability, race (or culture, ethnicity, or language), religion, sex, and sexual orientation. Age was the most frequently referenced characteristic (31/46; 67%) followed by race (or culture, ethnicity, or language; 25/46; 54%). Recommendations included specialist investigation and support for younger people affected by dementia and consideration of culture when assessing whether someone had dementia and providing person-centred care. Guidelines recommended considering religion when providing person-centred and end-of-life care. For disability, it was recommended that healthcare workers consider intellectual disability and sensory impairment when assessing for dementia. Most recommendations related to sex recommended not using sex hormones to treat cognitive impairment in men and women. One guideline made one recommendation related to sexual orientation. The main limitation of this study is that we only included national guidelines applicable to a whole country meaning guidelines from countries with differing healthcare systems within the country may have been excluded.

**Conclusions:**

National guidelines for dementia vary in their consideration of protected characteristics. We found that around a fifth of the world’s countries have guidelines for dementia. We have identified areas of good practice that can be considered for future guidelines and suggest that all guidelines provide specific evidence-based recommendations for minority groups with examples of how to implement them. This will promote equity in the care of people affected by dementia and help to ensure that people with protected characteristics also have high-quality clinical services.

## Abstract 2

Tiffeny James and team investigate which countries have national guidelines for dementia, and synthesise recommendations relating to protected characteristics as defined in the UK Equality Act 2010.

## Author summary

**Why was this study done?:**

- Clinical practice guidelines can improve the quality of dementia care, but some people with minority characteristics related, for example, to race or gender may be disadvantaged if the same approaches are used for everyone.
- It is not known whether national guidelines for dementia consider such characteristics in their recommendations.

**What did the researchers do and find?:**

- We did a systematic review searching in English and in the official languages of each country to identify which countries have national guidelines for dementia and assessed the extent to which they consider protected characteristics using the UK Equality Act 2010 to define protected characteristics.
- We identified 46 guidelines from 44 out of 196 countries in the world, most of which were high-income countries, and 37 of them included at least one recommendation related to protected characteristics including age, disability, race, religion, sex, and sexual orientation.
- Age and race were the most frequently referenced characteristics with recommendations including specialist investigation for younger people; consideration of culture and language when assessing dementia; and consideration of age, culture, and religion when providing person-centred care.

**What do these findings mean?:**

- We identified areas of good practice in higher-quality guidelines and recommend that all guidelines include specific evidence-based recommendations for minority groups, which are in line with the World Health Organisation’s human rights–based approach to dementia care.
- To our knowledge, this was the most comprehensive search for dementia guidelines to date; however, we only included national guidelines applicable to a whole country meaning that guidelines from countries with differing healthcare systems within the country have been excluded.

## Introduction

Around 50 million people live with dementia worldwide and numbers are predicted to increase to 152 million by 2050 [1]. As dementia is a global public health priority, the World Health Organisation (WHO) has developed a global action plan [2] monitored by the Global Dementia Observatory (GDO), which collates worldwide data on service delivery, research, and policies [3,4]. A key GDO indicator of a country’s dementia readiness is whether they have approved guidelines, protocols, or standards for dementia. Clinical practice guidelines provide evidence-based statements and recommendations to improve quality and consistency of care [5] and promote the most effective approaches to dementia care [6]. They should cover diagnosis, assessment, treatment, long-term care, and legal and ethical issues impacting the quality of care [1]. While guidelines aim to improve consistency of care, healthcare workers are encouraged to use judgement and adjust treatments to individual patient needs and preferences [5].

Health equity implies that everyone has a fair opportunity to achieve their full health potential and that no one is disadvantaged from achieving this [7,8], but certain groups may be disadvantaged. This often includes people with characteristics, which differ from the majority of people with dementia, such as people from minority ethnic groups who tend to present later to dementia services than the majority populations meaning diagnosis and treatment is delayed [9]. For people aged under 65, dementia diagnosis takes on average 4.4 years compared to 2.8 years for those over 65 suggesting that guidelines should address specific needs related to patient characteristics. In the United Kingdom, the Equality Act 2010 [10] describes 9 protected characteristics related to equity: age, disability, gender reassignment (referred to hereafter as gender identity to include persons whose gender may be different from their sex assigned at birth), marriage and civil partnership, pregnancy and maternity, race, religion or belief, sex, and sexual orientation. Adapting management for protected characteristics may help improve individualised treatment, but it is unclear to what extent dementia guidelines make recommendations about such characteristics.

Previous systematic reviews of dementia guidelines have synthesised recommendations of higher and moderate quality guidelines [11] and focused on specific areas of assessment or treatment [12–15]. Reviews have been mostly limited to English language guidelines using English-only search strategies [11,13,15], limiting their global reach. One analysis of English and German language dementia guidelines assessed the inclusion of ethical issues such as consideration of patient preferences and “seeing the patient as a person” [16], with results grouped together under wider headings like “adequate appreciation of the patient.” To our knowledge, no studies have assessed if and how guidelines for dementia consider minority or protected characteristics or make explicit recommendations to promote equity. This systematic review, therefore, aims to (1) identify which countries have official, national guidelines for dementia; and (2) synthesise guideline recommendations relating to age, disability, gender identity, marriage and civil partnership, pregnancy and maternity, race, religion or belief, sex, and sexual orientation.

## Methods

We followed PRISMA guidance (S1 Checklist), and we registered the review protocol on the International Prospective Register of Systematic Reviews: CRD4201916020 https://www.crd.york.ac.uk/prospero/display_record.php?RecordID=160205.

### Search strategy

We searched electronic databases (Cumulative Index to Nursing and Allied Health Literature (CINAHL), PsycINFO, and Medline) from inception to March 4, 2020, for Medical Subject Headings and keywords relating to dementia and guidelines (the search strategies are in S1 Appendix). We searched Guidelines International Network (GIN)‘s International Guideline Library on the same date using the word “dementia.” During preliminary exploratory searches using the internet search engine Google, we identified several guidelines not retrieved by either of these searches. To ensure the inclusion of these and other relevant guidelines, we did additional systematic searches using Google between April 21 and September 25, 2020, by searching for the phrase “dementia guideline” followed by each individual country name (*n =* 196) [17], for example, “dementia guideline Afghanistan”, “dementia guideline Albania”. We did the same for the phrase “Alzheimer’s guideline.”

We searched in English and in each country’s official languages, using Google Translate to translate the phrases [18]. For countries with more than 10 official languages, we searched only in English and in the language used by that country’s government if it was not English. If a language was not available on Google Translate, we searched only in English and in the official languages that were available. Eligible guidelines were nearly always found on the first page. We reviewed Google results until there were no relevant returns, which was typically no more than 5 pages. We also searched the governmental Department/Ministry of Health or similar website of each country for the words “dementia” and “Alzheimer” in English and in the official languages of that country if it was not English and browsed the content for guidelines using the available website menus. For government websites with no search function, we browsed the menus only. Some countries did not have an official government health website to enable a search. We checked our searches against the WHO GDO list of countries with approved guidelines, protocols, or standards for dementia.

### Eligibility criteria

We included official, national guidelines for the assessment, diagnosis, and management of dementia, which

- were about dementia in general, or included a section on dementia in guidelines for broader subjects, for example, Mental Health or Psychiatry;
- were applicable to a whole country and expected to be followed by healthcare workers;
- include recommendations;
- were developed, sponsored, or authorised at a national policy level such as a government Ministries, Departments, or Institutes of Health; and
- were from any country written in any language from any date.

We excluded guidelines that

- addressed only a specific aspect of dementia care;
- were not official, national guidelines for a country including those written by groups of researchers or clinicians or national or international Associations or Societies but not endorsed at a higher level; and
- summarised evidence on dementia care but did not include any recommendations.

### Study selection

#### Database searches

Two reviewers (TJ and HR) screened the titles and abstracts of a sample of 100 papers retrieved from CINAHL, PsycINFO, and Medline to standardise implementation of the eligibility criteria. We resolved disagreements about inclusion through discussion with a third researcher (GL) ensuring consistent screening, and TJ screened the remaining titles and abstracts independently, excluding those that were ineligible. Two reviewers (TJ and MK) screened the full text of all remaining papers. We agreed initially about inclusion for 60/66 (91%) papers and resolved disagreements (6/66; 9%) through discussion. We were unable to determine whether the remaining papers (*n =* 17) met the criteria from the guideline alone so we contacted guideline authors, international colleagues, dementia organisations, and Ministries of Health in the countries in question.

#### Guideline international network library

TJ excluded guidelines from GIN that were ineligible based on their titles only as there are no abstracts. Two reviewers (TJ and HR) screened the full texts of the remaining guidelines. We resolved the only disagreement about inclusion (1/17, 6%) through discussion. For 6 guidelines, eligibility was unclear, so we sought more information.

#### Individual country searches

TJ screened guidelines as part of the searching process. If there was more than one version of a guideline, we only included the most recent one, and when we identified summaries or reviews of guidelines, we sought the full versions and only included those.

### Quality assessment

We used the International Centre for Allied Health Evidence (iCAHE) Guideline Quality Checklist [19] to appraise the quality of included guidelines. We assessed quality of guidelines as a whole rather than individual recommendations. The iCAHE uses a binary response to address 14 items where “Yes” (1) indicates clear evidence of an item being addressed and “No” (0) indicates no clear evidence that an item has been addressed. We predetermined that we would classify guidelines as higher quality when there was clear evidence that they met the 4 following iCAHE guideline checklist criteria: (1) provides an outline of the search strategy used to find underlying evidence; (2) uses a hierarchy to rank the quality of underlying evidence; (3) appraises the quality of the evidence underpinning its recommendations; and (4) links the hierarchy and quality of underlying evidence to the recommendations. We classified all other guidelines as lower quality. Two reviewers (TJ and HR) assessed the quality of a sample of 40% (*n =* 18) eligible guidelines. We agreed on 229/252 (91%) criteria and resolved disagreements through discussion. TJ assessed the quality of the remaining 60% of guidelines independently. We prioritised recommendations from higher-quality guidelines.

### Data extraction and synthesis

Two reviewers independently extracted data from all eligible guidelines. We extracted recommendations and general text about the assessment, diagnosis, and treatment of dementia that related to the 9 protected characteristics defined by the UK Equality Act 2010. One reviewer (TJ) extracted data from all guidelines, using Google Translate to translate into English those written in other languages. The second reviewer was decided according to the language the guideline was written in (S2 Appendix). We recruited colleagues who work within Psychology or Psychiatry to help with data extraction of guidelines not written in English (see Acknowledgements for details). We created a matrix to indicate which countries have national guidelines for dementia according to our criteria and which protected characteristics they referenced either in their recommendations or general text. We provide a narrative synthesis of the recommendations we identified for each protected characteristic, prioritising recommendations from higher-quality guidelines.

## Results

### Study selection

**Fig 1:**
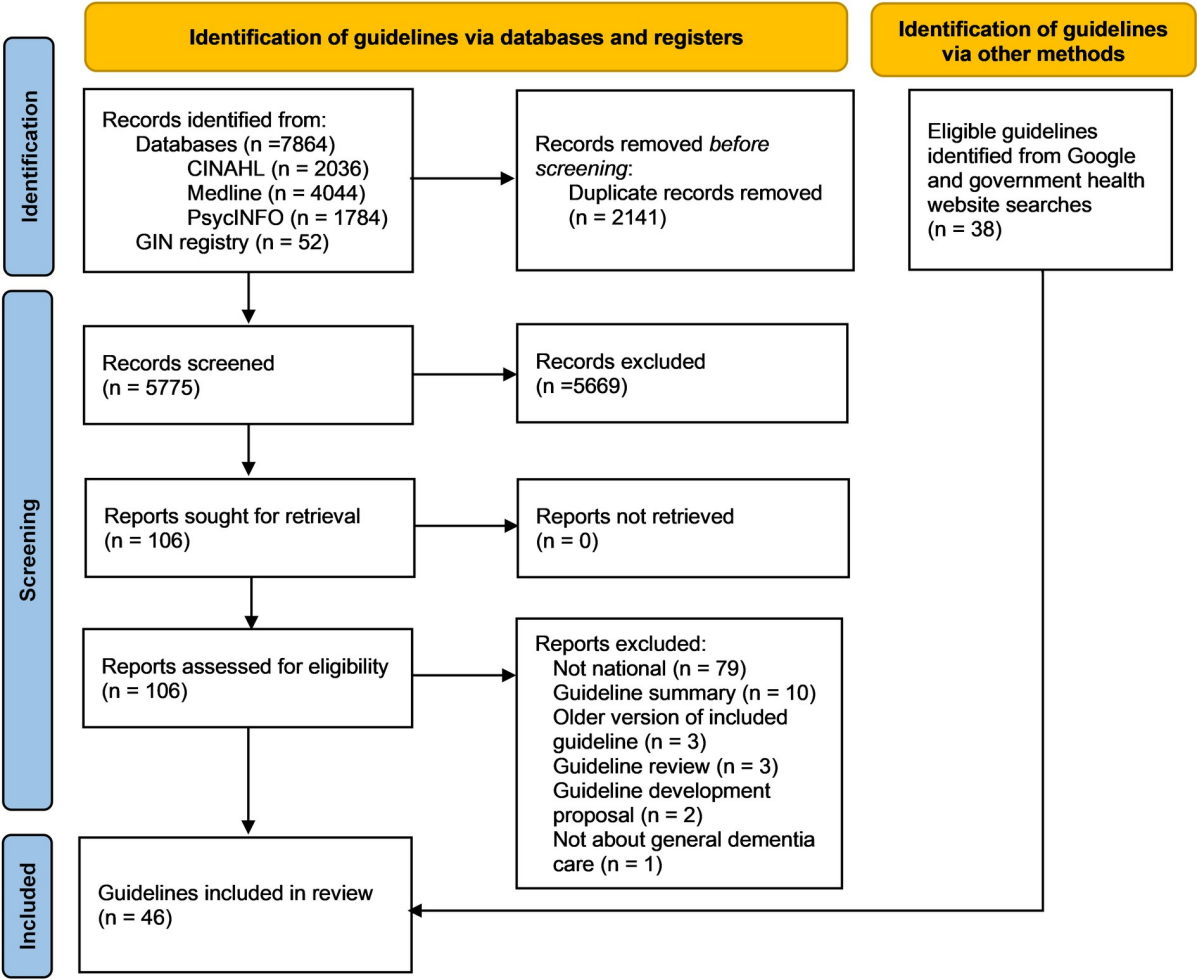
PRISMA flow diagram showing study selection. CINAHL, Cumulative Index to Nursing and Allied Health Literature; GIN, Guidelines International Network.

Forty-six guidelines from 44/196 (22%) countries met inclusion criteria (see Fig 1. PRISMA flow diagram). Mexico and Slovakia have separate guidelines for Alzheimer’s disease and vascular dementia. Dementia guidelines from Bahrain, Belarus, Ecuador, Macedonia, and Romania were part of larger guidelines for Psychiatry or Mental Health. We identified guidelines for 23/36 countries listed by the WHO GDO as having approved guidelines, protocols, or standards for dementia. We excluded documents for 13 countries listed by the WHO GDO [3] as they did not meet our criteria of either providing recommendations [20,21], being about dementia care generally [22,23], being approved at a national policy level [24,25], or being applicable to a whole country and expected to be followed by healthcare workers [26–34]. We did not find any relevant documents for Iran. We identified an additional 23 guidelines from 21 countries not listed by the WHO GDO. S2 Appendix shows where each guideline was found, and S3 Appendix describes the countries we were not able to do full searches for and the reasons.

### Quality assessment

We rated 18 (39%) guidelines as higher quality (S4 Appendix for full quality assessment). Higher quality guidelines were from Australia, Austria, Belgium, Colombia, Denmark, Finland, Germany, Japan, Malaysia, Mexico, Netherlands, Norway, Scotland, South Korea, Spain, Sweden, Switzerland, and the UK (excluding Scotland). The remaining guidelines (*n =* 28) were rated as lower quality (see Table 1). Only 18 (39%) guidelines provided an anticipated review date. Nine were in date [35–43], but for the other nine [44–52], the review date had passed at the time they were included in this review. Only 18 (39%) of guidelines provided an easily accessible summary of their recommendations [35,37,38,41–45,47,50,51,53–59].

**Table 1:** Guidelines and their country of origin, quality, and inclusion of recommendations related to protected characteristics.

Country	Year published	National organisation responsible for endorsing guideline (in English)	Quality	Age	Disability	Race, culture, ethnicity, or language	Religion	Sex	Sexual orientation	Listed on WHO GDO
Australia [35]	2016	NHMRC	H	+	+	+	+	/	O	✓
Austria [44]	2011	Competence Centre Integrated Care	H	+	/	+	/	+	/	✓
Belgium [37]	2017	National Institute for Health Disability Insurance	H	/	/	O	/	/	/	✓
Colombia [45]	2017	Ministry of Health and Social Protection	H	+	+	+	O	O	O	X
Denmark [54]	2019	National Board of Health	H	O	O	O	O	O	/	✓
Finland [38]	2011	Finnish Medical Society DUODECIM	H	O	+	/	/	/	/	✓
Germany [39]	2016	AWMF	H	+	/	+	/	+	/	✓
Japan [60]	2017	Ministry of Health, Labour and Welfare	H	O	/	/	/	/	/	✓
Malaysia [48]	2009	Ministry of Health	H	/	/	+	O	/	/	X
Mexico (AD) [41]	2017	CENETEC under Ministry of Health	H	+	/	+	/	/	/	X
Mexico (VaD) [42]	2017	CENETEC under Ministry of Health	H	/	/	/	/	/	/	X
the Netherlands [61]	2014	Federation of Medical Specialists	H	+	+	/	/	/	/	✓
Norway [56]	2017	Norwegian Directorate of Health	H	+	+	+	+	/	/	✓
Scotland [49]	2006	SIGN	H	+	/	/	/	+	/	X
Spain [51]	2010	Ministry of Health, Social Services and Equality	H	+	/	+	+	O	/	X
South Korea [62]	2011	Ministry of Health and Welfare	H	O	/	O	/	/	/	✓
Sweden [57]	2017	National Board of Health and Welfare	H	+	/	+	O	/	/	✓
United Kingdom [59]	2018	National Institute for Health and Clinical Excellence (NICE)	H	+	+	+	+	/	+	✓
Bahrain [53]	2004	Ministry of Health	L	+	/	O	/	/	/	X
Belarus [36]	2011	Ministry of Health	L	/	/	/	/	/	/	X
Brazil [63]	2017	Ministry of Health	L	+	/	/	/	/	/	✓
Chile [64]	2005	Ministry of Health	L	+	/	O	/	/	/	✓
Costa Rica [65]	2016	Ministry of Health	L	/	+	/	/	/	/	✓
Ecuador [66]	2008	Ministry of Public Health	L	/	/	/	/	/	/	X
France [67]	2011	High Authority of Health	L	+	/	+	/	/	/	✓
Georgia [46]	2009	Ministry of Health	L	/	/	/	/	/	/	X
Greece [68]	2014	Ministry of Health	L	O	+	+	/	/	/	✓
Hungary [69]	2008	Ministry of Health	L	+	+	/	O	/	/	✓
Israel [70]	2015	Ministry of Health	L	+	/	/	/	/	/	✓
Kazakhstan [40]	2015	Ministry of Health and Social Development	L	/	/	/	/	/	/	X
Latvia [55]	2017	National Health Service	L	+	+	/	/	+	/	X
Macedonia [47]	2004	Ministry of Health	L	/	/	/	/	+	/	X
New Zealand [71]	2013	Ministry of Health	L	+	+	+	+	/	O	X
Qatar [43]	2020	Ministry of Public Health	L	+	+	+	+	/	/	✓
Romania [72]	2010	Ministry of Health	L	/	/	/	/	+	/	X
Russia [73]	2013	Ministry of Health and Social Development	L	/	/	/	/	/	/	X
Serbia [74]	2013	Ministry of Health	L	+	/	O	O	+	/	X
Singapore [50]	2013	Ministry of Health	L	+	/	/	O	+	/	✓
Slovakia (AD) [75]	2020	Ministry of Health	L	+	/	/	/	/	/	X
Slovakia (VaD) [76]	2020	Ministry of Health	L	O	/	/	/	/	/	X
Switzerland [77]	2017	Swiss Academy of Medical Sciences	L	O	O	O	/	/	/	✓
Taiwan [78]	2017	Ministry of Health and Welfare	L	/	/	O	O	/	/	X
Thailand [79]	2014	Ministry of Public Health	L	+	O	/	+	/	/	✓
Turkey [58]	2020	Ministry of Health	L	/	/	O	/	/	/	X
Ukraine [52]	2016	Ministry of Health	L	+	+	+	+	/	/	X
Uruguay [80]	2015	Ministry of Public Heath	L	+	/	O	O	/	/	X

AD, Alzheimer’s disease; AWMF, Association of the Scientific Medical Societies in Germany; CENETEC, National Centre of Technological Excellence in Health; H, higher quality; L, lower quality; NHMRC, National Health and Medical Research Council; NICE, National Institute for Health and Clinical Excellence; SIGN, Scottish Intercollegiate Guidelines Network; VaD, vascular dementia; WHO GDO, World Health Organisation Global Dementia Observatory; +, has recommendations; O, no recommendations but mentions in main body of text; /, no recommendations or text; ✓, listed on WHO GDO; X, not listed on WHO GDO.

### Guidelines and recommendations

Table 1 describes guidelines, including ones identified in addition to those listed by the WHO GDO, and the protected characteristics considered in guidelines recommendations or text. We identified recommendations for 6 out of 9 protected characteristics: age, disability, race (or culture, ethnicity, or language), religion, sex, and sexual orientation. We did not identify any recommendations for gender identity, marriage and civil partnership, or pregnancy and maternity. Some guidelines discussed protected characteristics within the main body of text but did not include any related recommendations. Higher-quality guidelines from Austria, Australia, Colombia, Germany, Norway, Spain, and the UK provided recommendations relating to at least 3 protected characteristics. The UK guidelines included recommendations related to the most protected characteristics (5/6) but not sex. Guidelines from Denmark discussed all characteristics except sexual orientation in the text but did not provide any related recommendations. Lower-quality guidelines from Latvia, New Zealand, Qatar, and Ukraine made recommendations relating to at least 3 of the 6 protected characteristics. Seven (15%) guidelines made no recommendation or reference to protected characteristics at all; these were from Belarus, Ecuador, Georgia, Israel, Kazakhstan, Mexico (guidelines for vascular dementia), and Russia. Four guidelines made no recommendations relating to protected characteristics but referred in text to the protected characteristic of age (Japan and Slovakia for vascular dementia) or culture (Belgium and Turkey).

### Protected characteristics

#### Age

Twenty-five guidelines made recommendations relating to age [35,38,39,41,43–45,49–53,55–57,59,61,64,67,69,71,74,75,79], and 7 discussed age in the text but provided no recommendations [48,54,60,62,63,68,80]. Across higher- and lower-quality guidelines, the majority of recommendations regarding age were about young-onset dementia. Guidelines from Australia, Denmark, Norway, Japan, Singapore, Spain, Qatar, Ukraine, and the UK had a dedicated section on young-onset dementia. Belgian guidelines stated that the needs of people with young-onset dementia were outside the scope of the guideline, and Costa Rican guidelines specifically stated that there are no recommendations for young adults. Recommendations regarding age fell into 2 main categories:

**1) Specialist investigation for young-onset dementia**

Higher-quality guidelines recommended that people under the age of 60 are referred to specialist services for investigation (Norway, Finland, and Spain) or that specific tests are used to aid diagnosis. Austrian guidelines, for example, highlight the importance of structural imaging for people under the age of 60 and recommend use of either computed tomography or magnetic resonance imaging. Other guidelines recommend considering additional tests including cerebrospinal fluid or positron emission tomography scans (the Netherlands), and human immunodeficiency virus, syphilis, and drug screening (Germany), which are not recommended for assessment of dementia in people over the age of 60. UK guidelines recommend that healthcare workers are aware of potential genetic causes of young-onset dementia, and guidelines from Spain and Mexico (for Alzheimer’s disease) recommend genetic testing in younger people with a family history of young-onset dementia.

**2) Specialist support for people affected by young-onset dementia**

Higher-quality Australian, Colombian, Spanish, Swedish, and UK guidelines highlight the differing needs of people with young-onset dementia and recommend that service providers adapt their services to meet the needs of younger people and their families. For example, Australian guidelines consider younger people living with dementia in employment or caring for young children.

Recommendations and text relating to age in lower-quality guidelines was similar both in terms of specialist investigation [43,50,52,53,55,63,64,67–69,71,74,75,79,80] and support [43,50,52,68,71,76]. Other recommendations relating to age are that people should not be excluded from dementia services because of age (Colombia and Australia, higher quality; Greece and New Zealand, lower quality); that age should not be a contraindication for donepezil prescription for Alzheimer’s disease (Scotland, higher quality); and that as people’s wishes may change with age, they should be able to change their mind about decisions written in advanced directives (Spain, higher quality).

#### Summary

- Age was referenced in 31/46 (67%) guidelines.
- Recommendations around age typically relate to young-onset dementia, proposing
  - specialist investigation for young-onset dementia; and
  - specialist support for people affected by young-onset dementia.
- Nine guidelines had a dedicated section on young-onset dementia.

#### Disability

Twelve guidelines made recommendations regarding disability [35,38,43,45,52,55,56,59,61,65,68,71], and 5 discussed disability in the text without recommendations [54,58,69,77,79]. Most recommendations and text regarding disability were about sensory impairments and intellectual disabilities (IDs). Guidelines from Denmark, Malaysia Norway, Switzerland, Qatar, and Ukraine had sections about people with ID. There were 3 main types of recommendation in the higher-quality guidelines relating to disability:

1. people with ID and suspected dementia should be referred to specialist services for cognitive assessment (Denmark and Norway);
2. sensory impairment and ID should be considered when completing cognitive assessments and interpreting the scores (Australia, Colombia, Finland, and the UK); and
3. information about dementia should be provided in an accessible format to people living with dementia including people with ID or sensory impairment (Australia and Colombia).

The UK guidelines recommended that services should be accessible to people living with dementia including those with ID, sensory, and physical disabilities. Guidelines from Denmark included a small section highlighting the increased risk of dementia in people with ID but do not provide any recommendations. Recommendations or text regarding disability in lower-quality guidelines [43,52,55,65,68,69,71,77,79] were similar to higher-quality ones. Swiss guidelines have a dedicated section on ID but do not provide any recommendations.

#### Summary

- Disability was mentioned in 17/46 (37%) guidelines.
- Most recommendations refer to the potential impact of sensory impairment and ID on cognitive assessment scores.
- Six guidelines had a dedicated section on ID.

#### Sex

Eight guidelines had recommendations relating to sex [39,44,49,50,55,72,74,78], and 4 discussed sex within the text but made no recommendations [45,51,54,61]. The majority of recommendations were about the use of drug treatments for men or women specifically. Higher-quality guidelines from Scotland and Germany recommended not using hormone replacement therapy to treat symptoms of dementia in women, and higher-quality guidelines from Austria recommended not using androgens, such as testosterone, to treat Alzheimer’s disease in men. Within the text of higher-quality guidelines, Spain and Austria discussed a potentially higher care burden for women related to caring and domestic roles but made no recommendations about this. Dutch guidelines highlighted that men tend to overestimate their fitness to drive compared to objective tests and often drive for longer than they should, but there were no specific recommendations related to this.

Lower-quality guidelines also recommended not using hormone replacement therapy to treat cognitive decline [50,55]. Romanian and Serbian guidelines recommended the use of medroxyprogesterone to treat sexual disinhibition in men, with Serbian guidelines also recommending treatment with selective serotonin reuptake inhibitors (SSRIs) and carbamazepine to lower libido. In Taiwan, guidelines recommended letting patients touch or hold a doll as treatment of behavioural and psychiatric symptoms of dementia (BPSD), especially for female patients.

#### Summary

- Sex was referred to in 12/46 (26%) guidelines.
- Most of these recommendations suggested that sex hormones should not be used to treat cognitive impairment in men or women.
- Two lower-quality guidelines recommended the use of hormones or SSRIs to treat sexual disinhibition in men.

#### Sexual orientation

Four guidelines discussed sexual orientation, but the only specific recommendation was from the higher-quality UK guideline, which recommends that healthcare workers receive training on person-centred and outcome-focussed care such as consideration of a person’s sexuality. Higher-quality guidelines from Australia and Colombia discuss the need for healthcare providers to identify the diverse needs of people living with dementia including needs related to sexual orientation, but there are no recommendations related to this. Only one lower-quality guideline included reference to sexual orientation (New Zealand) but made no recommendations.

#### Summary

- Sexual orientation was mentioned in 4/46 (9%) guidelines.
- Only one guideline (UK) made one recommendation relating to sexual orientation, to consider sexual orientation when providing person-centred care.

#### Race, culture, ethnicity, or language

We include recommendations for the protected characteristic of race referring to the related concepts of race, culture, ethnicity, and language. We found no recommendations or text that used the term “race,” but guidelines referred to ethnic minority groups and personal diversity related to culture and language, which we refer to as culture in the remainder of the paper. Fifteen out of 46 guidelines made recommendations relating to culture [35,39,41,43–45,48,51,52,56,57,59,67,68,71], and 10 had text relating to culture but no recommendations [37,53,54,58,62,64,74,77,78,80]. Guidelines from Australia, Colombia, Denmark, New Zealand, Norway, and Ukraine had a specific chapter or subchapter about culture. Recommendations relating to culture from higher- and lower-quality guidelines concerned 2 main areas:

**1) Assessment of dementia and interpretation of scores**

In the higher-quality guidelines, recommendations included the consideration of culture and language when interpreting cognitive scores (Austria) and when choosing an appropriate assessment tool (Germany, Malaysia, and Spain). Guidelines from Australia and Sweden specifically recommend the use of the Rowland Universal Dementia Assessment Scale (RUDAS) for cognition, which is designed to be free from cultural effects [81], and the Mexican guidelines for Alzheimer’s disease recommend using the Reisberg Functional Assessment Staging Scale [82] to assess dementia as they suggest that it has greater cross-cultural validity. UK guidelines recommend that decisions about medication use according to dementia severity should not be determined on cognitive scores alone when there is no tool available in the patient’s language. Guidelines from Norway recommend that people from minority ethnic backgrounds are referred to specialist services if culture or language are barriers to assessment, and guidelines from Australia, Colombia, and Spain recommend the use of interpreters when conducting assessments and delivering information about diagnosis and treatment if necessary. Guidelines from Belgium, Denmark, and South Korea discussed the impact of culture and language on cognitive assessment but made no recommendations relating to this.

**2) Culture and person-centred care**

Higher-quality guidelines from Australia, Colombia, Norway, and the UK recommend that healthcare workers are trained in and provide person-centred care, which includes respecting a person’s culture. Swedish guidelines did not make recommendations about culture but provide examples of considering culture when providing person-centred care such as ensuring that people living with dementia have culturally adapted food and can access staff who speak their language. Malaysian guidelines discuss the need to consider culture when designing environments for people living with dementia. Australian guidelines recommend employing culturally diverse staff including Indigenous Australians to develop and deliver culturally sensitive dementia services including culturally appropriate care plans. UK and Colombian guidelines acknowledge the barriers that people from ethnic minority backgrounds face in accessing services, with UK guidance making specific recommendations that healthcare providers be aware of the need to ensure equality in access for people from ethnic minority backgrounds and that service providers design services to be accessible to people from diverse backgrounds.

Similarly, lower-quality guidelines stated that culture and language should be considered in the assessment of dementia and interpretation of scores and that appropriate tools should be used for assessment [43,52,58,64,67,68,71,74,80]. One lower-quality guideline from Ukraine recommended that healthcare workers be aware of the need to ensure equal access to people from different ethnic groups, and lower-quality guidelines from Bahrain, New Zealand, and Switzerland discussed the need to consider culture when delivering services and care.

#### Summary

- Race, culture, ethnicity, or language was mentioned in 25/46 (54%) of guidelines.
- Six guidelines had a dedicated section about culture.
- Recommendations around culture typically related to
  - the impact of culture on assessment of dementia and interpretation of scores; and
  - the importance of culture in person-centred care.

#### Religion

Eight guidelines included recommendations specific to religion [35,43,51,52,56,59,71,79], and 9 discussed religion in the text but provided no recommendations [45,48,50,54,57,69,74,78,80]. Under religion, we also include spirituality, as well as beliefs if used in a religious or spiritual context. Higher-quality guidelines from Australia, Spain, and the UK make recommendations related to religion. Australian guidelines recommended the consideration of religion when assessing behavioural and neuropsychological symptoms in people living with dementia, and UK guidelines recommend offering psychoeducation to carers of people living with dementia, which includes advice on emotional and spiritual well-being. Guidelines from Spain include a comprehensive section on end-of-life care, which recommends consideration of spiritual and religious factors. Guidelines from Denmark discuss the need to consider religion when providing end-of-life care but do not make specific recommendations. Guidelines from Norway recommend that care plans consider a person’s spiritual needs. Swedish guidelines highlight in text the need to ensure that people living with dementia have the opportunity to practice their religion as an example of how to provide culturally competent person-centred care but make no recommendations for this. Lower-quality guidelines also highlight the importance of religion when discussing end-of-life care [43,52,71,74,78–80] and treating behavioural and psychological symptoms of dementia [52,74].

#### Summary

- Seventeen out of 46 (37%) guidelines mentioned religion.
- Recommendations related to the importance of religion in
  - person-centred care;
  - end-of-life care; and
  - assessing and treating behavioural and psychological symptoms of dementia.

## Discussion

This study finds more national guidelines than previously listed [3], but we found only 44/196 (22%) countries had official, national guidelines for dementia, the majority being high-income countries. To our knowledge, this review is the first to assess national dementia guidelines for recommendations about the protected characteristics of age, disability, race, religion or belief, sex, and sexual orientation. We found that 85% of guidelines reference at least one of these characteristics either in their text or recommendations, but recommendations were often ambiguous or lacking sufficient evidence to back them up. Age was the most frequently referenced characteristic (32/46 guidelines) with typical recommendations that healthcare workers use specialist services and investigations to assess and care for people with dementia under the age of 60. Around half of guidelines discussed ethnicity, culture, or language, but the recommendations were vague. Several guidelines recommended using appropriate assessment tools for people who do not speak the local language but did not provide examples or lists of appropriate tools, instead relying on healthcare workers’ knowledge. Guidelines recommended that healthcare workers consider culture and religion when providing person-centred care, but very few gave examples of how to do this. There is a huge variation in guideline quality. Less than half of guidelines provided information on how evidence to support recommendations was gathered, prioritised, or appraised, raising questions about their integrity.

We only identified one recommendation relating to sexual orientation [59], and none related to gender identity. Older lesbian, gay, bisexual, and transgender (LGBT) adults have unique risks and needs associated with dementia, for example, often experiencing barriers to accessing dementia care or disclosing their sexual orientation or gender identity due to fear of discrimination [83,84]. Although healthcare workers report having affirming attitudes towards LGBT people with dementia [85], they lack knowledge and preparedness about the needs and management of this community [85,86]. Same-sex relationships are still illegal in some places, so we would not expect all countries to have related recommendations in their guidelines, but we still think it is important to aim for these rights for people living with dementia in all countries. With ageing populations and a gradual shift towards acceptance, the number of older adults who openly identify as LGBT is likely to increase, including people affected by dementia. Guidelines need to reflect this by providing specific recommendations that promote equity in care for LGBT people affected by dementia. While guidelines cannot give detailed recommendations relating to specific minority groups, they can provide general examples of how to provide equitable care related to protected characteristics, specify what should be considered, and signpost to appropriate guidance elsewhere.

Our review is in line with the WHO’s human rights–based approach for people living with dementia, which highlights the importance of nondiscrimination and equity in dementia care [87]. We used the UK Equality Act 2010 as a framework to operationalise equity in dementia guidelines as a standard to which many would aspire. However, we acknowledge that not all countries would apply the same standards for cultural, religious, or political reasons as with the example of same-sex relationships. We did not identify any recommendations related to pregnancy and maternity. This is not surprising as it is rare for people with dementia to be young enough to be pregnant as most people with young-onset dementia are over 50 [88]. We found no recommendations related to marriage and civil partnership. We think this may be because information about family carers who are often partners tends to be incorporated into all sections of guidelines rather than explicitly discussed as an individual characteristic. The right to partnership is important as is the right to be protected from a potential predatory partner, for example, one who wants the relationship in order to gain money or housing, when someone living with dementia has lost the ability to make judgements about a potential partner, and future guidelines could consider stating this explicitly.

To facilitate implementation, guidelines should provide a summary of their recommendations. Only around 40% of guidelines in this review did this, and several guidelines discussed protected characteristics in the main text but not in their recommendations. Guidelines are used in healthcare settings where quick access to information is valuable. Recommendations should be easily identifiable, and information about protected characteristics needs to be included in recommendations rather than in the main text where it is likely to be missed. While we assumed that the evidence-based quality of higher-quality clinical guidelines is higher, the quality of single recommendations may still differ. We included all recommendations in this review. Lower-quality guidelines from 2010 [72] and 2013 [74] recommend giving men drug treatments for sexual disinhibition without providing any evidence to support this. Although this recommendation considers the protected characteristic of sex, it may be considered ineffective, unsafe, or inequitable as may happen with any recommendations lacking sufficient or current evidence. Recommendations should be supported by current evidence, and guidelines should be updated frequently [89] or when new evidence indicates the need for this [5]. Available guidance on guideline development [5] and quality appraisal tools [19,90] should be updated to include guidance considering protected characteristics. These could be used to inform the development guidelines effective, equitable, and evidence-based recommendations.

Our review used a comprehensive search, translating search terms into the official languages of all countries, and included guidelines written in any language. Using this search strategy, we identified an additional 23 national guidelines from 21 countries that were not identified by the WHO GDO. We speculate that this is because WHO are informed by countries as to whether they have a national dementia policy, but that not all countries reply to enquiries or engage with the GDO. Development of the GDO is a dynamic process, and our findings may indicate a need to reach out and make better links with countries that have not replied as well as working with those who have not yet written guidelines. While this is the most comprehensive search for dementia guidelines to date, it took nearly a year to conduct them, translate potential guidelines, and collate the findings, meaning that the searches are only complete until September 2020. However, no new eligible or additional guidelines were identified in the 2021 update to the GDO database [3,91], suggesting that it is unlikely that we have missed any new national guidelines. Eligible guidelines still could have been missed due to a lack of local knowledge or alternative wordings used to describe them in other languages. We only included national guidelines applicable to a whole country meaning guidelines from parts of countries with differing healthcare systems within the country, such as the United States, have been excluded. The absence of unified guidelines in these settings may exacerbate existing health inequalities within these countries. There are also many other sets of recommendations from, for example, professionals organisations, which may be used by healthcare workers but did not fit our inclusion criteria. Two researchers extracted recommendations from all guidelines, and we recruited at least one researcher fluent in the language of the guideline, which minimised the chance of missing relevant recommendations.

The quality of services for people affected by dementia worldwide is improving, but it remains suboptimal in many places [91]. It is therefore not surprising that we only identified guidelines for 22% of countries and that some existing guidelines consider protected characteristics minimally or not at all. Countries wishing to develop national guidelines for dementia can adapt existing higher-quality ones to suit the local context rather than start from scratch [92]. Higher-quality Australian guidelines were adapted for Colombia and were one of the most comprehensive in terms of protected characteristics. Australian guidelines include sections about the needs of younger and culturally and linguistically diverse people living with dementia and were one of only 3 guidelines to recommend a specific test to assess cognitive function. Guidelines should recommend consideration of the effect of language on cognitive performance at the time of assessment and, ideally, use of a tool in the correct language with appropriate interpretation. Language can also be a huge barrier to receiving appropriate care and guidelines should recommend interpreters when conducting assessments and delivering information about diagnosis and treatment if necessary, as was done in Australian, Colombian, and Spanish guidelines. A small number of guidelines had dedicated sections on the characteristics of culture (*n =* 6), ID (*n* = 6), and age (*n* = 11), which are often associated with delays in diagnosis and, therefore, treatment of dementia. In our review, several guidelines adapted existing higher-quality guidelines without considering their own population’s characteristics. New or updated guidelines should consider how protected characteristics impact experiences of dementia within a population. These should include specific related recommendations across all aspects of dementia care from service access to assessment, treatment, and end-of-life care. This will help healthcare workers design tailored management strategies based on individual need.

## Conclusions

Existing national guidelines for dementia consider protected characteristics to varying degrees. While this review has highlighted a dearth of national guidelines for dementia, particularly in low- and middle-income countries, we have identified areas of good practice that can be considered for future guidelines and suggest that specific evidence-based recommendations for minority groups are made in all national policies for people, with gaps identified explicitly. Our findings apply to individual governments and health and social care services as well as people living with dementia and their families. Our results highlight the heterogeneity of coverage in national guidelines of important characteristics, which could be improved by using the best examples from individual health systems and policymakers about key priorities in dementia care and the evidence base for addressing these. Further research is needed to explore the experiences and needs of people affected by dementia who identify with different protected characteristics, for example, in LGBT communities who are underrepresented both in dementia research and guidelines, and this knowledge should be incorporated into future guideline recommendations.

## Supporting information

S1 Checklist PRISMA checklist. (DOCX)

S1 Appendix Search strategies. (DOCX)

S2 Appendix Language guidelines were written in and where they were found. (DOCX)

S3 Appendix Countries we were not able to search fully for reasons why and what we did. (DOCX)

S4 Appendix Quality assessment of included guidelines using iCAHE quality appraisal tool. (DOCX)

## Abbreviations

BPSD: behavioural and psychiatric symptoms of dementia
CINAHL: Cumulative Index to Nursing and Allied Health Literature
GDO: Global Dementia Observatory
GIN: Guidelines International Network
iCAHE: International Centre for Allied Health Evidence
ID: intellectual disability
LGBT: lesbian, gay, bisexual, and transgender
RUDAS: Rowland Universal Dementia Assessment Scale
SSRI: selective serotonin reuptake inhibitor
WHO: World Health Organisation
