# Supplementary material for: Protection against discrimination in national dementia guideline recommendations: A systematic review
Source: PLoS Med. 2022 Jan 11;19(1):e1003860. doi: 10.1371/journal.pmed.1003860 (PMC8752020; doi:10.1371/journal.pmed.1003860)
Supplement: S1 Checklist — (DOCX) [file pmed.1003860.s001.docx]

**S1 Checklist –** PRISMA checklist

| **Section and Topic** | **Item #** | **Checklist item** | **Location where item is reported** |
| --- | --- | --- | --- |
| **TITLE** | | |  |
| Title | 1 | Identify the report as a systematic review.  *Protection against discrimination in national dementia guideline recommendations: a systematic review.* | Title page |
| **ABSTRACT** | | |  |
| Abstract | 2 | See the PRISMA 2020 for Abstracts checklist. | Abstract section |
| **INTRODUCTION** | | |  |
| Rationale | 3 | Describe the rationale for the review in the context of existing knowledge.  *Adapting management for protected characteristics may help improve individualised treatment, but it is unclear to what extent dementia guidelines make recommendations about such characteristics.* | Introduction, paragraph 2 |
| Objectives | 4 | Provide an explicit statement of the objective(s) or question(s) the review addresses.  *This systematic review therefore aims to 1) identify which countries have official, national guidelines for dementia; and 2) synthesise guideline recommendations relating to age, disability, gender identity, marriage and civil partnership, pregnancy and maternity, race, religion or belief, sex, and sexual orientation.* | Introduction, paragraph 3 |
| **METHODS** | | |  |
| Eligibility criteria | 5 | Specify the inclusion and exclusion criteria for the review and how studies were grouped for the syntheses.  ***Eligibility criteria***  *We included official, national guidelines for the assessment, diagnosis, and management of dementia which:*   - *were about dementia in general, or included a section on dementia in guidelines for broader subjects e.g. Mental Health or Psychiatry* - *were applicable to a whole country and expected to be followed by healthcare workers* - *include recommendations* - *were developed, sponsored, or authorised at a national policy level such as a government Ministries, Departments, or Institutes of Health* - *were from any country written in any language from any date*   *We excluded guidelines which:*   - *addressed only a specific aspect of dementia care* - *were not official, national guidelines for a country including those written by groups of researchers or clinicians or national or international Associations or Societies but not endorsed at a higher level* - *summarised evidence on dementia care but did not include any recommendations*   *We provide a narrative synthesis of the recommendations we identified for each protected characteristic, prioritising recommendations from higher quality guidelines.* | Methods, paragraph 4 |
| Information sources | 6 | Specify all databases, registers, websites, organisations, reference lists and other sources searched or consulted to identify studies. Specify the date when each source was last searched or consulted.  *We searched electronic databases (Cumulative Index to Nursing and Allied Health Literature (CINAHL), PsycINFO and Medline) from inception to March 4^th^, 2020 for Medical Subject Headings and keywords relating to dementia and guidelines (the search strategy for Medline is in S1 Appendix). We searched Guidelines International Network (GIN)’s International Guideline Library on the same date using the word “dementia”. During preliminary exploratory searches using the internet search engine Google we identified several guidelines not retrieved by either of these searches. To ensure the inclusion of these and other relevant guidelines, we did additional systematic searches using Google between April 21^st^ and September 25^th^, 2020 by searching for the phrase “dementia guideline” followed by each individual country name (n = 196) [17] e.g. dementia guideline Afghanistan, dementia guideline Albania. We did the same for the phrase “Alzheimer guideline”.* | Methods, paragraph 2 |
| Search strategy | 7 | Present the full search strategies for all databases, registers and websites, including any filters and limits used.  **Search strategy**  *We searched electronic databases (Cumulative Index to Nursing and Allied Health Literature (CINAHL), PsycINFO and Medline) from inception to March 4^th^, 2020 for Medical Subject Headings and keywords relating to dementia and guidelines (the search strategies are in S1 Appendix)* | Methods, paragraph 2 |
| Selection process | 8 | Specify the methods used to decide whether a study met the inclusion criteria of the review, including how many reviewers screened each record and each report retrieved, whether they worked independently, and if applicable, details of automation tools used in the process.  ***Study selection***  *Database searches*  *Two reviewers (TJ and HR) screened the titles and abstracts of a sample of 100 papers retrieved from CINAHL, PsychINFO and Medline to standardise implementation of the eligibility criteria. We resolved disagreements about inclusion through discussion with a third researcher (GL) ensuring consistent screening and TJ screened the remaining titles and abstracts independently, excluding those that were ineligible. Two reviewers (TJ and MK) screened the full text of all remaining papers. We agreed initially about inclusion for 60/66 (91%) papers and resolved disagreements (6/66; 9%) through discussion. We were unable to determine whether the remaining papers (n = 17) met the criteria from the guideline alone so we contacted guideline authors; international colleagues; dementia organisations; and Ministries of Health in the countries in question.*  *Guideline international network library*  *TJ excluded guidelines from GIN that were ineligible based on their titles only as there are no abstracts. Two reviewers (TJ and HR) screened the full texts of the remaining guidelines. We resolved the only disagreement about inclusion (1/17, 6%) through discussion. For six guidelines eligibility was unclear, so we sought more information.*  *Individual country searches*  *TJ screened guidelines as part of the searching process. If there was more than one version of a guideline, we only included the most recent one and when we identified summaries or reviews of guidelines, we sought the full versions and only included those.* | Methods, paragraphs 5 – 7 |
| Data collection process | 9 | Specify the methods used to collect data from reports, including how many reviewers collected data from each report, whether they worked independently, any processes for obtaining or confirming data from study investigators, and if applicable, details of automation tools used in the process.  *Two reviewers independently extracted data from all eligible guidelines. One reviewer (TJ) extracted data from all guidelines, using Google Translate to translate into English those written in other languages. The second reviewer was decided according to the language the guideline was written in (S2 appendix) with colleagues helping with data extraction of guidelines not written in English.* | Methods, paragraph 9 |
| Data items | 10a | List and define all outcomes for which data were sought. Specify whether all results that were compatible with each outcome domain in each study were sought (e.g. for all measures, time points, analyses), and if not, the methods used to decide which results to collect.  *We extracted recommendations and general text about the assessment, diagnosis and treatment of dementia that related to the nine protected characteristics defined by the UK Equality Act 2010.* | Methods, paragraph 9 |
|  | 10b | List and define all other variables for which data were sought (e.g. participant and intervention characteristics, funding sources). Describe any assumptions made about any missing or unclear information.  *We created a matrix to indicate which countries have national guidelines for dementia according to our criteria and which protected characteristics they referenced either in their recommendations or general text.* | Methods, paragraph 9 |
| Study risk of bias assessment | 11 | Specify the methods used to assess risk of bias in the included studies, including details of the tool(s) used, how many reviewers assessed each study and whether they worked independently, and if applicable, details of automation tools used in the process.  *We used the International Centre for Allied Health Evidence (iCAHE) Guideline Quality Checklist [19] to appraise the quality of included guidelines. The iCAHE uses a binary response to address 14 items where ‘Yes’ (1) indicates clear evidence of an item being addressed and ‘No’ (0) indicates no clear evidence that an item has been addressed. We predetermined that we would classify guidelines as higher quality when there was clear evidence that they met the four following iCAHE guideline checklist criteria: 1) provides an outline of the search strategy used to find underlying evidence; 2) uses a hierarchy to rank the quality of underlying evidence; 3) appraises the quality of the evidence underpinning its recommendations; and 4) links the hierarchy and quality of underlying evidence to the recommendations. We classified all other guidelines as lower quality. Two reviewers (TJ and HR) assessed the quality of a sample of 40% (n = 18) eligible guidelines. We agreed on 229/252 (91%) criteria and resolved disagreements through discussion. TJ assessed the quality of the remaining 60% of guidelines independently. We prioritised recommendations from higher quality guidelines.* | Methods, paragraph 8 |
| Effect measures | 12 | Specify for each outcome the effect measure(s) (e.g. risk ratio, mean difference) used in the synthesis or presentation of results. | N/A |
| Synthesis methods | 13a | Describe the processes used to decide which studies were eligible for each synthesis (e.g. tabulating the study intervention characteristics and comparing against the planned groups for each synthesis (item #5)). | N/A |
|  | 13b | Describe any methods required to prepare the data for presentation or synthesis, such as handling of missing summary statistics, or data conversions. | N/A |
|  | 13c | Describe any methods used to tabulate or visually display results of individual studies and syntheses.  *We created a matrix to indicate which countries have national guidelines for dementia according to our criteria and which protected characteristics they referenced either in their recommendations or general text.* | Methods, paragraph 9 |
|  | 13d | Describe any methods used to synthesize results and provide a rationale for the choice(s). If meta-analysis was performed, describe the model(s), method(s) to identify the presence and extent of statistical heterogeneity, and software package(s) used. | N/A |
|  | 13e | Describe any methods used to explore possible causes of heterogeneity among study results (e.g. subgroup analysis, meta-regression). | N/A |
|  | 13f | Describe any sensitivity analyses conducted to assess robustness of the synthesized results. | N/A |
| Reporting bias assessment | 14 | Describe any methods used to assess risk of bias due to missing results in a synthesis (arising from reporting biases). | N/A |
| Certainty assessment | 15 | Describe any methods used to assess certainty (or confidence) in the body of evidence for an outcome. | N/A |
| **RESULTS** | | |  |
| Study selection | 16a | Describe the results of the search and selection process, from the number of records identified in the search to the number of studies included in the review, ideally using a flow diagram.  ***Study selection***  *Forty six guidelines from 44/196 (22%) countries met inclusion criteria (see fig.1 PRISMA flow diagram). Mexico and Slovakia have separate guidelines for Alzheimer’s disease and vascular dementia. Dementia guidelines from Bahrain, Belarus, Ecuador, Macedonia, and Romania were part of larger guidelines for Psychiatry or Mental Health. We identified guidelines for 23/36 countries listed by the WHO GDO as having approved guidelines, protocols, or standards for dementia.* | Results, paragraph 1 |
|  | 16b | Cite studies that might appear to meet the inclusion criteria, but which were excluded, and explain why they were excluded.  *We excluded documents for 13 countries listed by the WHO GDO [3] as they did not meet our criteria of either providing recommendations [20, 21]; being about dementia care generally [22, 23] being approved at a national policy level [24, 25]; or being applicable to a whole country and expected to be followed by healthcare workers [26-34]. We did not find any relevant documents for Iran. We identified an additional 23 guidelines from 21 countries not listed by the WHO GDO. S2 Appendix shows where each guideline was found and S3 Appendix describes the countries we were not able to do full searches for and the reasons.* | Results, paragraph 1 |
| Study characteristics | 17 | Cite each included study and present its characteristics.  *Table 1* | Results, – Table 1 |
| Risk of bias in studies | 18 | Present assessments of risk of bias for each included study  *Quality assessment*  *We rated 18 (39%) guidelines as higher quality (S4 appendix for full quality assessment). Higher quality guidelines were from Australia, Austria, Belgium, Colombia, Denmark, Finland, Germany, Japan, Malaysia, Mexico, Netherlands, Norway, Scotland, South Korea, Spain, Sweden, Switzerland, and the UK (excluding Scotland). The remaining guidelines (n = 28) were rated as lower quality (see table 1). Only 18 (39%) guidelines provided an anticipated review date. Nine were in date [35-43] but for the other nine [44-52], the review date had passed at the time they were included in this review. Only 18 (39%) of guidelines provided an easily accessible summary of their recommendations [35, 37, 38, 41-45, 47, 50, 51, 53-59].*  Description of study quality is in the results, paragraph 2  Guideline quality (higher or lower) in table 1 in results section  Full quality assessment is in S4 Appendix: quality assessment of included guidelines using iCAHE quality appraisal tool. | Various – see left hand column |
| Results of individual studies | 19 | For all outcomes, present, for each study: (a) summary statistics for each group (where appropriate) and (b) an effect estimate and its precision (e.g. confidence/credible interval), ideally using structured tables or plots. | N/A |
| Results of syntheses | 20a | For each synthesis, briefly summarise the characteristics and risk of bias among contributing studies.  *Characteristics and quality of guidelines are discussed per protected characteristic* | Results, paragraph 4 onwards |
|  | 20b | Present results of all statistical syntheses conducted. If meta-analysis was done, present for each the summary estimate and its precision (e.g. confidence/credible interval) and measures of statistical heterogeneity. If comparing groups, describe the direction of the effect. | N/A |
|  | 20c | Present results of all investigations of possible causes of heterogeneity among study results. | N/A |
|  | 20d | Present results of all sensitivity analyses conducted to assess the robustness of the synthesized results. | N/A |
| Reporting biases | 21 | Present assessments of risk of bias due to missing results (arising from reporting biases) for each synthesis assessed. | N/A |
| Certainty of evidence | 22 | Present assessments of certainty (or confidence) in the body of evidence for each outcome assessed. | N/A |
| **DISCUSSION** | | |  |
| Discussion | 23a | Provide a general interpretation of the results in the context of other evidence.  *This study finds more national guidelines than previously listed [3] but we found only 44/196 (22%) countries had official, national guidelines for dementia, the majority being high-income countries. To our knowledge, this review is the first to assess national dementia guidelines for recommendations about the protected characteristics of age, disability, race, religion or belief, sex, and sexual orientation. We found that 85% of guidelines reference at least one of these characteristics either in their text or recommendations, but recommendations were often ambiguous or lacking sufficient evidence to back them up. Age was the most frequently referenced characteristic (32/46 guidelines) with typical recommendations that healthcare workers use specialist services and investigations to assess and care for people with dementia under the age of 60. Around half of guidelines discussed ethnicity, culture, or language but the recommendations were vague. Several guidelines recommended using “appropriate assessment tools” for people who do not speak the local language but did not provide examples or lists of appropriate tools, instead relying on healthcare workers’ knowledge. Guidelines recommended that healthcare workers consider culture and religion when providing person centred care, but very few gave examples of how to do this. There is a huge variation in guideline quality. Less than half of guidelines provided information on how evidence to support recommendations was gathered, prioritised, or appraised, raising questions about their integrity.* | Discussion, paragraph 1. |
|  | 23b | Discuss any limitations of the evidence included in the review.  *To facilitate implementation, guidelines should provide a summary of their recommendations. Only around 40% of guidelines in this review did this, and several guidelines discussed protected characteristics in the main text but not in their recommendations*  *Lower-quality guidelines from 2010 [72] and 2013 [74] recommend giving men drug treatments for sexual disinhibition without providing any evidence to support this. Although this recommendation considers the protected characteristic of sex, it may be considered ineffective, unsafe, or inequitable as may happen with any recommendations lacking sufficient or current evidence.*  *In our review, several guidelines adapted existing higher quality guidelines without considering their own population’s characteristics* | Discussion, paragraph 4  Discussion, paragraph 4  Discussion section, paragraph 6 |
|  | 23c | Discuss any limitations of the review processes used.  We used the UK Equality Act 2010 as a framework to operationalise equity in dementia guidelines as a standard to which many would aspire. However, we acknowledge that not all countries would apply the same standards for cultural, religious, or political reasons as with the example of same-sex relationships.  While we assumed that the evidence-based quality of higher quality clinical guidelines is higher, the quality of single recommendations may still differ  Whilst this is the most comprehensive search for dementia guidelines to date, it took nearly a year to conduct them and collate the findings, meaning the searches are only complete until September 2020. However, no new eligible guidelines were identified in the 2021 update to the GDO database [3, 91] suggesting it is unlikely that we have missed any new national guidelines. Eligible guidelines still could have been missed due to a lack of local knowledge or alternative wordings used to describe them in other languages. We only included national guidelines applicable to a whole country meaning guidelines from countries with differing healthcare systems within the country, such as the United States, may have been excluded.  There are also many other sets of recommendations from, for example, professionals organisations which may be used by healthcare workers but did not fit our inclusion criteria. | Discussion, paragraph 3  Discussion, paragraph 4  Discussion, paragraph 5  Discussion, paragraph 5 |
|  | 23d | Discuss implications of the results for practice, policy, and future research.  *Same-sex relationships are still illegal in some places so we would not expect all countries to have related recommendations in their guidelines, but we still think it is important to aim for these rights for people living with dementia in all countries. With aging populations and a gradual shift towards acceptance, the number of older adults who openly identify as LGBT is likely to increase, including people affected by dementia. Guidelines need to reflect this by providing specific recommendations that promote equity in care for LGBT people affected by dementia. Whilst guidelines cannot give detailed recommendations relating to specific minority groups, they can provide general examples of how to provide equitable care related to protected characteristics, specify what should be considered, and signpost to appropriate guidance elsewhere.*  *To facilitate implementation, guidelines should provide a summary of their recommendations. Only around 40% of guidelines in this review did this, and several guidelines discussed protected characteristics in the main text but not in their recommendations. Guidelines are used in healthcare settings where quick access to information is valuable. Recommendations should be easily identifiable, and information about protected characteristics needs to be included in recommendations rather than in the main text where it is likely to be missed. We included all recommendations in this review.*  *Using this search strategy, we identified an additional 23 national guidelines from 21 countries which were not identified by the WHO GDO. We speculate that this is because the WHO are informed by countries what is done, but that not all countries reply to enquiries or engage with the GDO. Development of the GDO is a dynamic process and our findings may indicate a need to reach out and make better links with countries that have not replied as well as working with those who have not yet written guidelines*  *Recommendations should be supported by current evidence, and guidelines should be updated frequently [89] or when new evidence indicates the need for this [5]. Available guidance on guideline development [5] and quality appraisal tools [19, 90] should be updated to include guidance considering protected characteristics. These could be used to inform the development guidelines effective, equitable and evidence-based recommendations.*  *New or updated guidelines should consider how protected characteristics impact experiences of dementia within a population. These should include specific related recommendations across all aspects of dementia care from service access to assessment, treatment, and end-of-life* *care. This will help healthcare workers design tailored management strategies based on individual need.* | Discussion, paragraph 2  Discussion, paragraph 4  Discussion, paragraph 5  Discussion, paragraph 4  Discussion, paragraph 6 |
| **OTHER INFORMATION** | | |  |
| Registration and protocol | 24a | Provide registration information for the review, including register name and registration number, or state that the review was not registered.  We registered the review protocol on the International Prospective Register of Systematic Reviews: CRD4201916020 <https://www.crd.york.ac.uk/prospero/display_record.php?RecordID=160205> | Methods, paragraph 1 |
|  | 24b | Indicate where the review protocol can be accessed, or state that a protocol was not prepared.  We registered the review protocol on the International Prospective Register of Systematic Reviews: CRD4201916020 <https://www.crd.york.ac.uk/prospero/display_record.php?RecordID=160205> | Methods, paragraph 1 |
|  | 24c | Describe and explain any amendments to information provided at registration or in the protocol. | N/A |
| Support | 25 | Describe sources of financial or non-financial support for the review, and the role of the funders or sponsors in the review.  *This report is independent research funded by the National Institute for Health Research (NIHR) Applied Research Collaboration (ARC) North Thames. The views expressed in this publication are those of the authors and not necessarily those of the NIHR or the Department of Health and Social Care. Award number NIHR 200163 (TJ). https://www.arc-nt.nihr.ac.uk/. The funders had no role in study design, data collection and analysis, decision to publish, or preparation of the manuscript. GL is supported by University College London Hospitals’ (UCLH) NIHR Biomedical Research Centre ARC North Thames and as an NIHR Senior Investigator. AS is funded by the UCLH NIHR Biomedical Research Centre. NM is funded by an Alzheimer’s Society Senior Fellowship.* | Not included in paper as requested by editor |
| Competing interests | 26 | Declare any competing interests of review authors.  *Declaration of conflicts of interest: none.* | Not included in paper as requested by editor |
| Availability of data, code and other materials | 27 | Report which of the following are publicly available and where they can be found: template data collection forms; data extracted from included studies; data used for all analyses; analytic code; any other materials used in the review. | N/A |
