# Supplementary material for: Protection against discrimination in national dementia guideline recommendations: A systematic review
Source: PLoS Med. 2022 Jan 11;19(1):e1003860. doi: 10.1371/journal.pmed.1003860 (PMC8752020; doi:10.1371/journal.pmed.1003860)
Supplement: S1 Appendix — (DOCX) [file pmed.1003860.s002.docx]

**S1 Appendix** - Search strategies

*Medline*

| 1 exp Dementia [MeSH] |
| --- |
| 2 dementia* |
| 3 alzheimer* |
| 4 exp Guideline [MeSH] |
| 5 guideline* [Text word] |
| 6 (clinical adj2 guideline*) |
| 7 (national adj1 guideline*) |
| 8 (practice adj guideline*) |
| 9 1 or 2 or 3 |
| 10 4 or 5 or 6 or 7 or 8 |
| 11 9 and 10 |

MeSH = Medical Subject Heading

| 1 exp Dementia [MeSH] |
| --- |
| 2 dementia* |
| 3 alzheimer* |
| 4 exp Treatment Guideline [MeSH] |
| 5 guideline* |
| 6 (clinical adj2 guideline*) |
| 7 (national adj1 guideline*) |
| 8 (practice adj guideline*) |
| 9 1 or 2 or 3 |
| 10 4 or 5 or 6 or 7 or 8 |
| 11 9 and 10 |

*PsycINFO*

MeSH = Medical Subject Heading

*CINAHL*

| 1 exp Dementia [MeSH] |
| --- |
| 2 dementia* |
| 3 alzheimer* |
| 4 exp Practice Guideline [MeSH] |
| 5 guideline* [Text word] |
| 6 (clinical n2 guideline*) |
| 7 (national n1 guideline*) |
| 8 (practice n1 guideline*) |
| 9 1 or 2 or 3 |
| 10 4 or 5 or 6 or 7 or 8 |
| 11 9 and 10 |

MeSH = Medical Subject Heading
