# Supplementary material for: Protection against discrimination in national dementia guideline recommendations: A systematic review
Source: PLoS Med. 2022 Jan 11;19(1):e1003860. doi: 10.1371/journal.pmed.1003860 (PMC8752020; doi:10.1371/journal.pmed.1003860)
Supplement: S2 Appendix — (DOCX) [file pmed.1003860.s003.docx]

**S2 Appendix** – Language guidelines were written in and where they were found.

| **Country** | **Language guideline written in** | **Where guideline was found** | **Search phrase (if applicable)** |
| --- | --- | --- | --- |
| Australia | English | Medline | N/A |
| Austria | German | Google | “dementia guideline Germany” in German |
| Bahrain | English | Google | “dementia guideline Bahrain” in Arabic |
| Belarus | Russian | Google | “dementia guidelines Belarus” in Russian |
| Belgium | Dutch | GIN | N/A |
| Brazil | Portuguese | Google | “dementia guideline Brazil” in Portuguese |
| Chile | Spanish | Google | “dementia guideline Chile” in Spanish |
| Colombia | Spanish | Google | “dementia guideline Colombia” in Spanish |
| Denmark | Danish | Google | “dementia guideline Denmark” in English |
| Ecuador | Spanish | Google | “dementia guidelines Colombia” in Spanish |
| Finland | Finnish | Google | “dementia guideline Finland” in Finnish |
| France | French | Medline | N/A |
| Georgia | Georgian | Google | “dementia guideline Georgia” in Georgian |
| Germany | German | PsycINFO | N/A |
| Greece | Greek | Google | “dementia guideline Greece” in Greek |
| Hungary | Hungarian | Google | “dementia guideline Hungary” in Hungarian |
| Israel | Hebrew | Google | “dementia guideline Israel” in Hebrew |
| Japan | Japanese | Google | “dementia guideline Japan” in Japanese |
| Kazakhstan | Russian | Google | “dementia guideline Kazakhstan” in Russian |
| Latvia | Latvian | Google | “dementia guideline Latvia” in Latvian |
| Macedonia | Macedonian | Google | “dementia guideline Macedonia” in Macedonian |
| Malaysia | English | GIN | N/A |
| Mexico (AD) | Spanish | Google | “dementia guideline Mexico” in Spanish |
| Mexico (VaD) | Spanish | Google | “dementia guideline Mexico” in Spanish |
| Netherlands | Dutch | Google | “dementia guideline Netherlands” in Dutch |
| New Zealand | English | Google | “dementia guideline New Zealand” in English |
| Norway | Norwegian | Google | “dementia guideline Norway” in Norwegian |
| Qatar | English | Ministry of Health | “dementia” in English |
| Romania | Romanian | Google | “dementia guideline Romania” in Romanian |
| Russia | Russian | Google | “dementia guideline Russia” in Russian |
| Scotland | English | GIN | N/A |
| Serbia | Serbian | Google | “dementia guideline Servia” in Serbian |
| Singapore | English | Google | “dementia guideline Singapore” in English |
| Slovakia (AD) | Slovak | Ministry of Health | “dementia” in Slovak |
| Slovakia (VaD) | Slovak | Ministry of Health | “dementia” in Slovak |
| South Korea | Korean | Google | “dementia guideline South Korea” in English |
| Spain | Spanish (English version also available) | Google | “dementia guideline Spain” in English |
| Sweden | Swedish | Google | “dementia guideline Sweden” in Swedish |
| Switzerland | German (English version also available) | Medline | N/A |
| Taiwan | Chinese | Google | “dementia guideline Taiwan” in Chinese |
| Thailand | Thai | Google | “dementia guideline Thailand” in Thai |
| Turkey | Turkish | Ministry of Health | “dementia” in Turkish |
| UK | English | Medline | N/A |
| Ukraine | Ukrainian | Colleague* | N/A |
| Uruguay | Spanish | Google | “dementia guideline Uruguay” in Spanish |

GIN = Guideline International Network; AD = Alzheimer’s Disease; VaD = Vascular dementia. *A Ukrainian document was retrieved by GIN searches but a Ukrainian colleague confirmed that this was not eligible and sent us the official, national guidelines for dementia which we included in this review.
