# Supplementary material for: Protection against discrimination in national dementia guideline recommendations: A systematic review
Source: PLoS Med. 2022 Jan 11;19(1):e1003860. doi: 10.1371/journal.pmed.1003860 (PMC8752020; doi:10.1371/journal.pmed.1003860)
Supplement: S3 Appendix — (DOCX) [file pmed.1003860.s004.docx]

**S3 Appendix** – Countries we were not able to search fully for, reasons why, and what we did.

| **Country** | **Google Translate not available for these languages:** | **No search function on government health website** | **Government health website inaccessible** |
| --- | --- | --- | --- |
| Afghanistan | Dari |  |  |
| Albania |  |  | ✓* |
| Algeria |  | ✓ |  |
| Antigua and Barbuda |  | ✓ |  |
| Armenia |  | ✓ |  |
| Bhutan | Dzongkha |  |  |
| Bolivia | Has 36 languages – we searched in English and in Spanish (used in government) |  |  |
| Bosnia and Herzegovina |  | ✓ |  |
| Burundi | Burundi |  |  |
| Côte d'Ivoire |  | ✓ |  |
| Central African Republic | Sango |  |  |
| Chad |  | ✓ |  |
| Comoros | Comorian |  | ✓* |
| Djibouti |  | ✓ |  |
| Egypt |  |  | ✓ |
| Ethiopia |  | ✓ |  |
| Fiji | Fijian and Fijian Hindi |  |  |
| Gabon |  |  | ✓ |
| Ghana |  | ✓ |  |
| Grenada |  | ✓ |  |
| Guatemala |  | ✓ |  |
| Guyana |  | ✓ |  |
| Honduras |  | ✓ |  |
| India | Has 22 languages – we searched in English and Hindi (used in government) | ✓ |  |
| Indonesia |  |  | ✓ |
| Iran |  |  | ✓ |
| Iraq |  | ✓ |  |
| Kiribati | Kiribati | ✓ |  |
| Kuwait |  | ✓ |  |
| Laos |  | ✓ |  |
| Lesotho |  | ✓ |  |
| Maldives | Dhivehi |  |  |
| Marshall Islands | Marshallese |  | ✓* |
| Mexico | Has 69 languages – we searched in English and Spanish (used in government) |  | ✓* |
| Micronesia |  |  | ✓ |
| Montenegro | Montenegrin |  |  |
| Mozambique |  | ✓ |  |
| Namibia |  | ✓ |  |
| Nauru | Nauruan |  |  |
| Nepal |  | ✓ |  |
| Palau | Palauan | ✓ |  |
| Palestine state |  | ✓ |  |
| Papa New Guinea | Tok Pisin and Hiri Motu | ✓ |  |
| Saudi Arabia |  | ✓ |  |
| Seychelles | Seselwa | ✓ |  |
| Syria |  |  | ✓ |
| Tanzania |  | ✓ |  |
| Timor-Leste | Tetum |  |  |
| Togo |  |  | ✓* |
| Tonga |  | ✓ |  |
| Turkmenistan |  | ✓ |  |
| Tuvalu | Tuvaluan |  | ✓* |
| Vanuatu | Bismala |  |  |
| Venezuela |  | ✓ |  |
| Zimbabwe | Has 16 languages – we searched in English (used in government) |  |  |

✓* no government health website.
